# Direct and indirect determinants of childhood malaria morbidity in Malawi: a survey cross-sectional analysis based on malaria indicator survey data for 2012

**DOI:** 10.1186/s12936-015-0777-1

**Published:** 2015-07-08

**Authors:** Simangaliso Chitunhu, Eustasius Musenge

**Affiliations:** Division of Epidemiology and Biostatistics, School of Public Health, Faculty of Health Sciences, University of the Witwatersrand, 27 St Andrews’ Road, Parktown, Johannesburg, 2193 South Africa

**Keywords:** Childhood malaria, Direct determinant, Indirect determinant, Propensity score matching, Structural equation modelling

## Abstract

**Background:**

Children under the age of five are most vulnerable to malaria (malaria is a major health challenge in sub-Saharan Africa) with a child dying every 30 s from malaria. Hampered socio-economic development, poverty, diseconomies of scale, marginalization, and exploitation are associated with malaria. Therefore establishing determinants of malaria in affected sub-Saharan populations is important in order to come up with informed interventions that will be effective in malaria control.

**Methods:**

The study was a cross-sectional survey design based on data from the Malawi 2012 Malaria indicator Survey obtained from Demographic and Health Survey (DHS) programme website. The outcome variable was positive laboratory-based blood smear result for malaria in children less than 5 years, after an initial positive rapid malaria diagnostic test done at the homestead. Statistical modelling was done using survey logistic regression as well as generalized structural equation modelling (G-SEM) to analyse direct and indirect effects of malaria.

**Results:**

The propensity score matched data had 1 325 children with 367 (27.7%) having blood smear positive malaria. Female children made up approximately 53% of the total study participants. Child related variables (age, haemoglobin and position in household) and household wealth index were significant directly and indirectly. Further on G-SEM based multivariable analysis showed socio-economic status (SES) [Odds ratio (OR) = 0.96, 95% Confidence interval (CI) = 0.92, 0.99] and primary level of education [OR = 0.50, 95% CI = 0.32, 0.77] were important direct and indirect determinants of malaria morbidity.

**Conclusion:**

Socio-economic status and education are important factors that influence malaria control. These factors need to be taken into consideration when planning malaria control programmes in order to have effective programmes. Direct and indirect effect modelling can also provide an alternative modelling technique that incorporates surrogate confounders that may not be significant when modelled directly. This holistic approach is useful and will help in improving malaria control.

## Background

Malaria is a common cause of ill-health and death mainly in the poorest countries of the world [1]. Most poor countries in sub-Saharan Africa are affected, with nine out of ten cases of the global malaria morbidity [2]. This makes it one of the most important global health problems [3]. Malaria is endemic and a major public health problem to Malawi, a low income country in sub-Saharan Africa [2, 4]. In the year 2010, malaria accounted for the third highest number of deaths in Malawi [5–7].

Malaria is caused by Plasmodium parasites and transmitted through the bite of Anopheles mosquitoes [8]. Transmission is mainly determined by climatic factors: temperature, humidity, and rainfall [9–12]. Other factors that also determine transmission include socio-economic status, knowledge on malaria prevention methods as well as access to health services [13–16]. The extent and distribution of these factors influence the prevalence rate. Transmission is highest in areas of high temperature and frequent rainfall, especially in summer [17–20].

Malaria is a disease and cause of poverty and has determinants of vulnerability [21, 22], because poor communities cannot afford malaria prevention tools, treatment tools and housing that is protective from mosquitoes [23]. According to a report on the epidemiological profile of malaria and its control in Malawi [24], the country is low-income and is amongst one of the poorest nations of the world. Poverty levels are extremely high with about 65% of the population being classified as poor [25]. In 2012, Malawi was classified as one of the ten poorest countries in the world [26] and urbanization is very low [24]. Therefore, there is need to fully understand the determinants of malaria in order to reduce the burden that malaria puts on the health care system [27, 28] due to the poverty levels in Malawi. Identifying direct and indirect determinants of malaria in a low income malaria endemic country will assist in the identification of important determinants of the disease and this will help in the development of health programmes that target those determinants in order to effectively reduce the burden of malaria with the available resources and also inform health policy [29]. Policies that the poor countries can afford are important as they may be easier to implement [30].

The aim of this study was to investigate the direct and indirect determinants of malaria morbidity in children under 5 years using pathway analysis on data from the Malawi malaria indicator survey collected in 2012. This age group was selected as it is the most vulnerable in malaria endemic areas [31–34].

## Methods

### Study area

Malawi is a country in southern Africa that has an area of approximately 120,000 km^2^ and is bordered by Zambia to the west, Mozambique to the south and Tanzania to the north of the country [4]. The presence of many water bodies especially on the eastern side makes the nation vulnerable to malaria morbidity and mortality.

### Malawi malaria indicator survey data

The malaria data used for the analysis were obtained from the 2012 malaria indicator survey (MIS) and were obtained from the Demographic and Health Survey (DHS) programme website. The original study collected data on basic demographic and health indicators, malaria prevention, treatment and morbidity. A total of 3,500 households were selected for data collection. A two-stage cluster sampling technique was used to select the households. The first stage selected 140 enumeration areas (EAs) with 96 from rural areas and 44 from urban areas. At the second stage, 25 households per EA were selected. The data were obtained through use of a household questionnaire that collected household characteristics, and identified all household members and their basic characteristics. Data for children less than five were collected from their mothers. Population sampling adjustments weights were done for the 140 clusters (EAs) to account for differences due to the unequal proportions selected per cluster [4, 35]. Malaria in children under five was initially tested at the household using a rapid malaria diagnostic test and those who tested positive had their blood collected for a confirmatory blood smear laboratory test [4, 35]. A positive blood smear laboratory test was used as the main outcome variable in data analysis. The variables used were region, type of place of residence, cluster altitude, wealth index of household, position of child in the family, child’s age in month, use of bed net on the previous night before the interview, mother’s knowledge of malaria, mother’s level of education, child’s altitude-adjusted haemoglobin level and time to get to the source of water. The wealth index of a household is a measure of the household’s standard of living and is based on data on household’s ownership of durable goods, dwelling characteristics, water source, toilet facilities, and other characteristics that are indicators of a household’s socioeconomic status [4, 35]. A special variable was created and used as proxy for socio-economic status. It was based on presence of tap water, toilet and electricity in the household. Presence of all three was defined as none slum and absence of one or more of these variables was defined as slum. This was based on a study that was done in rural South Africa [36].

### Statistical analysis methods

The sample size was determined during the primary study; it was established that data used in this study had a greater than 80% power. Since the data were observational, propensity score matching on some unbalanced selected variables was done to adjust for selection bias [37, 38].

As a proxy for use of preventative methods the variable on use of bed net the previous night by children under five was utilized. This was then used as the treatment variable and propensity scores were extracted post multivariable logistic regression. The method that was used in matching these scores was caliper matching [39].

Survey adjusted bivariate analysis was done using Pearson’s Chi Square and Student’s t-tests. Variables that were selected for multivariable analysis were based on their significant association with the outcome variable. Smear positive malaria result was modelled using survey logistic regression in order to determine the associations between the independent variables that were selected for analysis. Clustered robust method was used in analysing the data and the cluster was the primary sampling unit also used as the weighting variable. Generalized structural equation modelling (G-SEM) was used to model the direct and indirect pathways. This direct and an indirect model was developed to analyse the complex relationships between selected variables and the pathways that were conceptualized as having had an impact on a child having a smear positive malaria result in a household in 2012. All statistical analyses for this paper were carried out using Stata^®^13.1 (Copyright 1985–2013, StataCorp LP).

### Ethics approval

This study was granted ethics approval by the University of the Witwatersrand’s Human Research Ethics Committee (Medical) (Clearance Certificate No. M130962). Approval to use the MIS data was obtained from the measure DHS website. The primary study, where the data was collected, verbal informed consent for testing of children was obtained from the child’s parent or guardian at the end of the household interview and ethical clearances with the Malawi authorities before study started. The survey was also anonymized so that household or individual information is not identifiable [35].

## Results

The total number of children used in the study was 1,898 and their ages ranged from 6 months to 59 months with a mean age of 32.06 months. This was the number of children who were tested for malaria using a laboratory-based test, of whom 468 (24.7%) had a positive result for malaria and 1,430 (75.3%) had a negative result for malaria. The Central Province had 53.3% of the total cases; the Southern Province had 37.4% and the Northern Province had 9.3% of total cases. A total of 522 (27.1%) children were from urban areas and 1 376 (72.9%) were from the rural areas. Female children made up approximately 53% of the total study participants. Most of the mothers in this study had no education (71.7%), but 55.4% of the mothers were able to read whole sentences.

In the propensity-matched data, a total of 1,325 children were analysed with 367 (27.7%) having blood smear positive malaria and 958 (72.3%) having no malaria. Table 1 shows the descriptive statistics for both matched and unmatched data that were selected for analysis looking at the association between the selected variable and positive blood smear for malaria. An association was considered significant if it had a *p* value of less than 0.05. Univariate and multiple variable analyses were done to establish the relationships between blood smear positive malaria and selected variables and how they influence blood smear positive malaria in children under 5 years of age.

**Table 1:** Descriptive statistics of both initial and propensity score matched data for Malawi in 2012

Independent variables		Unmatched data			Propensity score matched data		
Variable	Category	Blood smear positive [n = 468 (24.7%)]	Blood smear negative [n = 1430 (74.9%)]	Test statistic (p-value^a^)	Blood smear positive [n = 367 (27.7%)]	Blood smear negative [n = 958 (72.3%)]	Test statistic (p-value)
Region*	Northern	60 (9.3%)	266(15.4%)	χ^2^ = 3.64 (0.01)	47 (9.6%)	176 (15.4%)	χ^2^ = 3.27 (0.015)
	Central	234 (53.3%)	525 (38.0%)		181 (51.4%)	348 (36.6%)	
	Southern	174 (37.4%)	639 (46.6%)		139 (39.0%)	434 (48.1%)	
Type of place of residence	Urban	54 (5.0%)	468 (16.4%)	χ^2^ = 9.82 (<0.01)	48 (5.9%)	278 (14.3%)	χ^2^ = 5.96 (0.003)
	Rural	414 (95.0%)	962 (83.6%)		319 (94.1%)	680 (85.7%)	
Cluster altitude (km)	Mean ± SE	0.90 ± 0.03	0.89 ± 0.03	t = −0.49 (0.623)	0.90 ± 0.03	0.88 ± 0.03	t = −0.43 (0.667)
Position of child in household	1	254 (54.1%)	973 (68.0%)	χ^2^ = 11.17 (<0.01)	224 (59.8%)	723 (74.3%)	χ^2^ = 7.20 (<0.001)
	2	153 (32.6%)	297 (20.2%)		135 (38.1%)	217 (24.0%)	
	3	61 (13.3%)	160 (11.8%)		8 (2.1%)	18 (1.7%)	
Mother’s highest education level*	None	415 (88.9%)	949 (73.1%)	χ^2^ = 7.30 (< 0.01)	329 (89.6%)	700 (78.8%)	χ^2^ = 3.54 (0.012)
	Primary	47 (9.9%)	361 (21.5%)		34 (9.3%)	221 (18.9%)	
	Secondary	6 (1.2%)	120 (5.4%)		4 (1.1%)	37 (2.3%)	
Mother has heard of malaria	No	47 (10.5%)	62 (4.9%)	χ^2^ = 4.27 (0.02)	38 (11.0%)	50 (5.9%)	χ^2^ = 3.09 (0.048)
	Yes	421 (89.5%)	1,368 (95.1%)		329 (89.0%)	908 (94.1%)	
Child’s age in months*	Mean ± SE	34.79 ± 0.56	31.25 ± 0.40	t = −5.08 (<0.001)	34.7 ± 0.64	31.7 ± 0.52	t = −3.60 (<0.001)
Child’s altitude adjusted haemoglobin level	Mean ± SE	9.2 ± 0.96	10.4 ± 0.56	t = 11.90 (<0.01)	9.2 ± 0.91	10.3 ± 0.63	t = 10.7 (<0.001
Wealth index score*	Mean ± SE	−5.58 ± 0.25	−2.47 ± 0.32	t = 8.05 (< 0.01)	−5.68 ± 0.35	−3.56 ± 0.36	t = 6.41 (<0.001)
Children under 5 slept under mosquito bed net last night*	No	206 (41.3%)	488 (34.1%)	χ^2^ = 1.89 (0.15)	184 (47.4%)	481 (48.8%)	χ^2^ = 0.36 (0.691)
	Yes	262 (58.7%)	942 (65.9%)		183 (52.6%)	477 (51.2%)	
Time in hours to get to water source*	Mean ± SE	5.23 ± 0.52	6.63 ± 0.47	t = 2.71 (< 0.01)	5.05 ± 0.55	6.18 ± 0.49	t = 1.92 (0.057)

*SE* standard error.

* Variables that were used in propensity score matching.

^a^significance was calculated at 5%.

Table 2 shows results of the univariate survey logistic regression, multiple variable survey logistic regression as well as the results of the G-SEM. Table 3 shows the results of the propensity score matched results of the same data.

**Table 2:** Univariate, multiple variable and G-SEM analyses of results of unmatched data for children 6–59 months in Malawi in 2012

Variable	Univariate analysis	Multivariable analysis
	Odds ratio (95% CI), p-value	Odds ratio (95% CI), p-value
Region		
Northern	1.00	1.00
Central	2.43 (1.24, 4.74), 0.01	1.79 (1.24, 2.59), <0.01
Southern	1.36 (0.69, 2.68), 0.89	0.89 (0.58, 1.39), 0.62
Type of place of residence		
Urban	1.00	1.00
Rural	3.87 (2.22, 6.73), <0.01	1.83 (1.18, 2.83), <0.01
Cluster altitude in kilometers	1.11 (0.63, 1.96), 0.73	0.72 (0.45, 1.12), 0.15
Child’s position in household		
1	1.00	1.00
2	2.03 (1.59, 2.60), <0.01	1.43 (1.04, 1.96), 0.03
3	1.46 (1.08, 1.99), 0.02	0.99 (0.40, 2.45), 0.98
Mother’s highest education level		
None	1.00	1.00
Primary	0.40 (0.28, 0.57), <0.01	0.53 (0.37, 0.76), <0.01
Secondary	0.18 (0.08, 0.42), <0.01	0.57 (0.23, 1.47), 0.25
Child’s age in months	1.02 (1.00, 1.02), <0.01	1.03 (1.02, 1.04), <0.01
Child’s haemoglobin level	0.95 (0.95, 0.96), <0.01	0.95 (0.94, 0.96), <0.01
Wealth index score	0.90 (0.87, 0.94), <0.01	0.95 (0.93, 0.98), <0.01
Child slept under mosquito bed net		
No	1.00	1.00
Yes	0.74 (0.54, 1.03), 0.07	0.77 (0.60, 0.99), 0.04
Time to water source	0.98 (0.96, 1.00), 0.01	0.97 (0.96, 0.99), <0.01

**Table 3:** Propensity score matched univariate and multivariable results for children 6–59 months in Malawi in 2012

Variable category	Univariate analysis	Multivariable analysis	G-SEM direct effects	G-SEM indirect effects
	Odds ratio (95% CI), p-value	Odds ratio (95% CI), p-value	Odds ratio (95% CI), p-value	Odds ratio (95% CI), p-value
Region				
Northern	1.00	1.00	1.00	
Central	2.33 (1.22, 4.48), 0.01	1.92 (1.26, 2.92), <0.01	1.92 (1.03, 3.55), 0.04	
Southern	1.32 (0.69, 2.56), 0.394	0.96 (0.58, 1.59), 0.88	0.96 (0.48, 1.91), 0.91	
Type of place of residence				
Urban	1.00	1.00	1.00	
Rural	2.74 (1.55, 4.88), <0.01	1.58 (0.97, 2.56), 0.07	1.58 (0.84, 2.94), 0.15	
Cluster altitude in kilometers	1.11 (0.59, 2.06), 0.75	0.75 (0.44, 1.29), 0.30	0.75 (0.31, 1.84), 0.53	1.24 (0.87, 1.62), <0.01
Position of child in household				
1	1.00	1.00	1.00	1.03 (1.01, 1.05), <0.01
2	1.91 (1.39, 2.63), <0.01	1.49 (1.05, 2.10), 0.02	1.49 (1.04, 2.14), 0.03	
3	1.73 (0.73, 4.09), 0.21	1.12 (0.41, 3.08), 0.82	1.12 (0.42, 2.99), 0.82	
Child’s age in months	1.01 (1.01, 1.02), <0.01	1.03 (1.02, 1.04), <0.01	1.03 (1.02, 1.04), <0.01	
Child’s altitude adjusted haemoglobin level	0.95 (0.94, 0.96), < 0.01	0.94 (0.93, 0.95), < 0.01	0.94 (0.93, 0.95), <0.01	
Mother’s highest education level				
None	1.00	1.00	1.00	0.50 (0.28, 0.71), <0.01
Primary	0.45 (0.31, 0.66), <0.01	0.50 (0.32, 0.77), <0.01	0.50 (0.32, 0.76), <0.01	
Secondary	0.40 (0.14, 1.19), 0.10	0.71 (0.20, 2.52), 0.60	0.71 (0.19, 2.71), 0.62	
Wealth index score	0.91 (0.88, 0.95), <0.01	0.96 (0.92, 0.99), 0.01	0.96 (0.92, 0.99), 0.01	
Children slept under mosquito bed net				
No	1.00	1.00	1.00	
Yes	1.07 (0.77, 1.50), 0.69	0.77 (0.58, 1.01), 0.06	0.77 (0.56, 1.04), 0.09	
Time to get to water source	0.98 (0.96, 1.00), 0.07	0.97 (0.95, 0.99), <0.01	0.97 (0.95, 0.99), <0.01	

These results from logistic regression model as well as the generalized structural equation modelling show that socio-economic status (SES) represented by wealth index; region, time to water source, mother’s highest level of education, child haemoglobin level (OR = 0.95, CI = 0.94, 0.96) as well as child’s age were important determinants of malaria episodes in children aged between 6 and 59 months in Malawi in the year 2012. Age also showed (OR = 1.03, CI = 1.02, 1.04) that positive blood smear malaria increased with increasing age and the analysis on the position of the child showed that a child in the second position was almost one and half times likely (OR = 1.43, CI = 1.04, 1.96) as a child in first position to get malaria. Time to water source was also significant in this study (OR = 0.97, CI = 0.96–0.99).

In this study, type of place of residence (urban or rural) also showed a significant effect on malaria. Those who stay in the rural areas were more likely to have a positive blood smear result for malaria as compared to their counterparts in the urban areas (OR = 1.83, CI = 1.18, 2.83). Region of residence was also an important factor in this study (p = < 0.01). The central region of Malawi was the most affected with a 79% greater odds of malaria morbidity compared to the northern and southern regions [1.79 (CI = 1.24, 2.59)].

**Figure 1:**
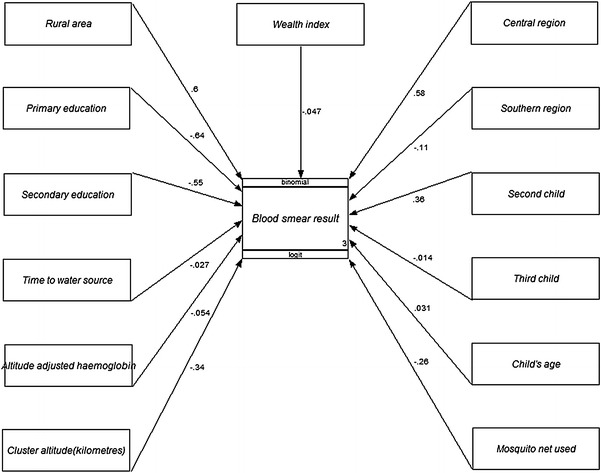
G-SEM path diagram showing coefficients from binomial logistic regression analysis of the effects of selected random variables on a blood smear positive malaria result in children under five in Malawi in 2012.

**Figure 2:**
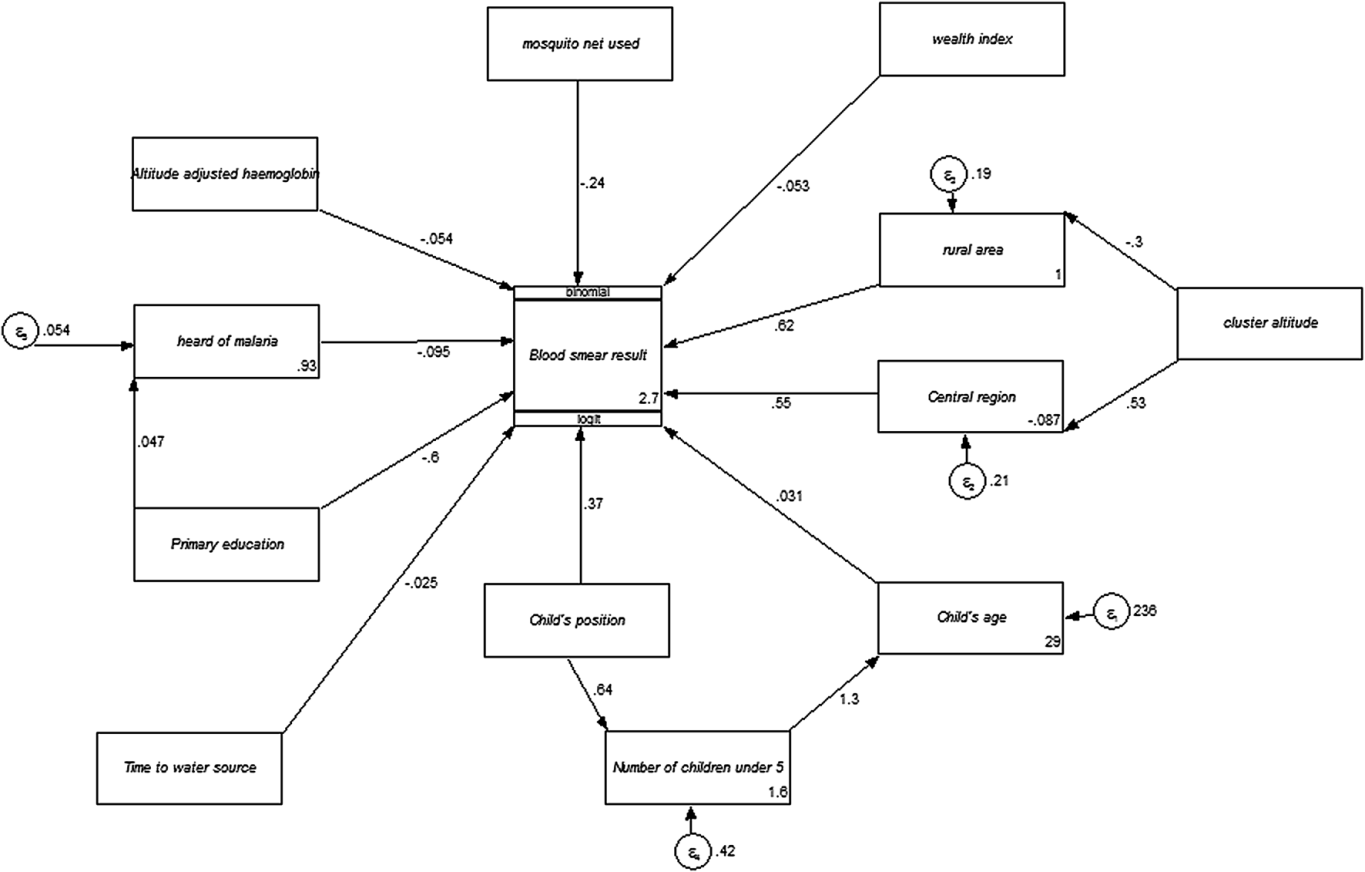
G-SEM path diagram of selected random variables showing both direct and indirect pathways related to blood smear positive malaria results for children less than 5 years in Malawi in 2012.

The results of the G-SEM show both direct and indirect effects on the endogenous variable blood smear positive malaria. Figures 1 and 2 show the G-SEM models. Figure 1 showing the direct G-SEM and Figure 2 showing the indirect G-SEM. Exogenous variables; rural area means type of place of residence and primary education represents mother’s level of education.

The indirect effects were modelled on the variable cluster altitude, mother’s highest education level and the wealth index score. Cluster altitude and knowledge of malaria were identified as variables that can indirectly affect blood smear positive malaria in children less than five.

## Discussion

SES is an important determinant of malaria and other studies [21, 23, 40] also showed that SES is an important factor in blood smear positive malaria. As mentioned earlier malaria is a disease of poverty [21, 41] so this finding compliments what other studies have shown with regards to this particular variable on malaria morbidity. One review article on economic and social burden of malaria stated that malaria thrives in poor countries [42]. The results from this study support this review because Malawi has a low GDP [43]; this means malaria puts an extra burden on the government as well as an extra burden on the population in terms of accessing healthcare. The government needs to ensure that the resources are available for diagnosis as well as treatment and the population must have the necessary financial means in order to access the treatment [42]. If the population cannot afford this treatment, then the government is forced to provide the treatment at affordable costs and this might affect the government’s self sufficiency. Since malaria is endemic in Malawi; the government needs to put in place measures to control malaria. These include providing insecticide-treated mosquito nets, indoor residual spraying as well as providing anti-malarial tablets [44] and these activities strain the budget of the country and other economic building activities will not be prioritized and therefore this affects the overall development of the country. In order to balance the spending on malaria treatment and the spending on other developmental activities, it is important for the country to know the malaria trends so that the use of resources is optimized.

GDP also affects nutrition, because nutrition is linked to economic status if one is economically sound then they are able to provide adequately for themselves and, therefore, resulting in a well-nourished body. A well-nourished body is better able to fight malaria infection by mounting an adequate response to infection as compared to an undernourished body [45]. Health status is also linked to economic status and malaria is also affected by the economic status of an individual as well as country [46]. A poor economic status results in inadequate health care facilities and therefore increasing vulnerability of the population to malaria.

Type of place of residence as well as region could be linked to the altitude, where some studies [47–49] found an effect on malaria prevalence depending on the altitude. It is important for the government to know the areas that are malaria “hotspots” so that malaria prevention resources can be allocated to the areas that have higher malaria morbidity as compared to the rest of the country. Malaria endemicity is also influenced by temperature and rainfall and altitude also influences temperature and rainfall [50, 51], this is explored in further work using spatial modelling.

Education level of the mother also showed significance (p ≤ 0.001). The results showed that the more educated a mother was, the less likely the child was to have blood smear positive malaria. This could be due to the fact that an educated mother better understands information on malaria and are more likely to implement properly the preventive measures that they are taught. And also that educated mothers a more likely to be employed or be entrepreneurs, hence getting income to sustain the family and children better.

Child related variables (haemoglobin level, position of child and age of child) were also significant in influencing blood smear positive malaria in children under five. Studies [19, 52–54] have shown that anaemia is a complication of malaria so this study confirms this and children with a low haemoglobin level had higher chances of having malaria as compared to children with normal haemoglobin levels From the descriptive statistics only 32.3% were not anemic and this might be linked to the low SES resulting in poor nutrition [45]. Water bodies especially stagnant water sources [50, 51] are known as breeding places for mosquitoes, therefore, this study showed that those who were nearer to water sources were at a higher risk for malaria.

The G-SEM’s indirect pathways also showed a significant association between cluster altitude and region as well as between SES and education level. G-SEM was used in this study to complement the results from the multiple variable analysis and the results showed that the multiple variable analysis and the G-SEM direct pathways show similar results. G-SEM can help in diagrammatically conceptualizing the effects of the determinants on the outcome and this helps in analysis where the variables can then be separated into those with a direct effect on the outcome and those with an indirect effect on the outcome. This will help to explain better some factors that might not directly affect the outcome, and inform policy on adopting indirect and direct approaches to dealing with the disease in children.

Some of the study limitations were that the data could not be verified and was used as it was and this might have limited the analysis as well. Multi-collinearity was another limitation of the study as some variables could not be used due this problem and were dropped from the multivariable analysis, resulting in loss of useful information and might have affected the interpretation of the results. In this study, data from a cross sectional study was used so although the study was looking at blood smear positive malaria, cross-sectional studies mainly measure prevalence and not incidence. This, therefore, limited the interpretation of the associations that were observed during data analysis. There was no temporal sequence that could be ascertained from this type of study design.

Strengths of the study were the use of propensity score matching in order to deal with selection bias and ultimately confounding. Matching was done based on insecticide treated net usage as this activity had the potential of confounding the outcome. The outcome was based on a rapid diagnostic test result as well as a laboratory test result for malaria so this was strength of the study in that the outcome was based on laboratory confirmed results and not affected by recall bias. Survey-adjusted multiple logistic regression as well as structural equation modelling were used in analysis to cater well for direct and indirect determinants.

## Conclusions

It is important to understand the determinants of malaria so that effective monitoring and evaluation of malaria can be carried out. This study showed the importance of socio-economic status as well as education in the fight against malaria. In order for malaria to be eliminated in the population it is important for the government to empower the population economically and also ensure that health education is a part of the efforts that are put in place to fight malaria. This will assist in the fight to eliminate malaria. It is important to ensure that resources are channeled in order to optimize prevention strategies that are put in place. Once the population is empowered, then preventative strategies for malaria elimination can then be implemented successfully and if the population is educated, then it is able to understand better the strategies in place and implement them successfully. The other important determinates also are linked to socio-economic status, therefore, reduction of poverty will go a long way in the fight to eliminate malaria.

## Abbreviations

CI: confidence interval
DHS: demographic and health survey
DST: Department of Science and Technology
EA: enumeration area
GDP: gross domestic product
G-SEM: generalized-structural equation modelling
HIV: human immunodeficiency virus
ITNs: insecticide-treated bed nets
MDGs: millennium development goals
MIS: malaria indicator survey
NRF: National Research Foundation
OR: odds ratio
SACEMA: South African Centre of Excellence in Epidemiological Modelling and Analysis
SES: socio-economic status
WHO: World Health Organization
